# Update in inclusion body myositis

**DOI:** 10.1097/01.bor.0000434671.77891.9a

**Published:** 2013-09-25

**Authors:** Pedro Machado, Stefen Brady, Michael G. Hanna

**Affiliations:** MRC Centre for Neuromuscular Diseases, Institute of Neurology, University College London, London, UK; ∗ Pedro Machado and Stefen Brady have contributed equally to this article.

**Keywords:** diagnosis, inclusion body myositis, myopathies, pathogenesis, treatment

## Abstract

**Purpose of review:**

The purpose of this study is to review recent scientific advances relating to the natural history, cause, treatment and serum and imaging biomarkers of inclusion body myositis (IBM).

**Recent findings:**

Several theories regarding the aetiopathogenesis of IBM are being explored and new therapeutic approaches are being investigated. New diagnostic criteria have been proposed, reflecting the knowledge that the diagnostic pathological findings may be absent in patients with clinically typical IBM. The role of MRI in IBM is expanding and knowledge about pathological biomarkers is increasing. The recent description of autoantibodies to cytosolic 5′ nucleotidase 1A in patients with IBM is a potentially important advance that may aid early diagnosis and provides new evidence regarding the role of autoimmunity in IBM.

**Summary:**

IBM remains an enigmatic and often misdiagnosed disease. The pathogenesis of the disease is still not fully understood. To date, pharmacological treatment trials have failed to show clear efficacy. Future research should continue to focus on improving understanding of the pathophysiological mechanisms of the disease and on the identification of reliable and sensitive outcome measures for clinical trials. IBM is a rare disease and international multicentre collaboration for trials is important to translate research advances into improved patient outcomes.

## INTRODUCTION

Sporadic inclusion body myositis (IBM) is the commonest acquired myopathy in patients aged over 50 years [1]. It is classified along with polymyositis, dermatomyositis and immune-mediated necrotizing myopathies as an idiopathic inflammatory myopathy. However, IBM is distinguished from these other disorders by asymmetric finger flexor and knee extensor weakness [2] and resistance to immunosuppressive therapy [3]. Several pathological findings on muscle biopsy are considered as synonymous with the diagnosis of IBM: an endomysial inflammatory infiltrate, invasion of nonnecrotic muscle fibres by inflammatory cells (partial invasion), rimmed vacuoles, amyloid and 15–18 nm tubulofilaments on electron microscopy. Combinations of these features have formed the basis of successive diagnostic criteria for IBM [4–7]. However, these histological findings may not all be present in patients with a clinically typical IBM [3,8]. This is reflected by the inclusion of a clinically defined group in the new 2011 European Neuromuscular Centre (ENMC) diagnostic criteria [9], which build on the MRC Centre criteria [10,11]. New developments include the description of autoantibodies to cytosolic 5′ nucleotidase 1A (cN1A) in patients with IBM [12,13^▪▪^,^14^^▪▪^]. Two recently published studies [13^▪▪^,^14^^▪▪^] assess their diagnostic use in IBM. This review focuses on our current knowledge of IBM with particular emphasis on developments in the last 24 months in disease, serum and imaging biomarkers and on on-going and future therapeutic trials.

## NATURAL HISTORY STUDIES

Recent studies [3,15,16^▪^,^17^] investigating the natural history of IBM have confirmed the typical early disease phenotype and highlighted that IBM is often initially misdiagnosed, polymyositis being the most common incorrect initial diagnosis. They have also shown that survival in patients with IBM seems to be similar to the general population, but late-stage disease can cause very significant morbidity, including disability and reduced quality of life. Death in IBM is related to malnutrition, cachexia, aspiration, respiratory infection and respiratory failure, as a consequence of dysphagia, severe global weakness and weakness of the respiratory muscles [3,17]. In a Dutch cohort [17], euthanasia or continuous deep sedation was used by 13% of patients with IBM, in comparison with 20% of patients with amyotrophic lateral sclerosis [12]. These data highlight the morbidity experienced by IBM patients and the importance of supportive and palliative care in IBM.

**Box 1:**
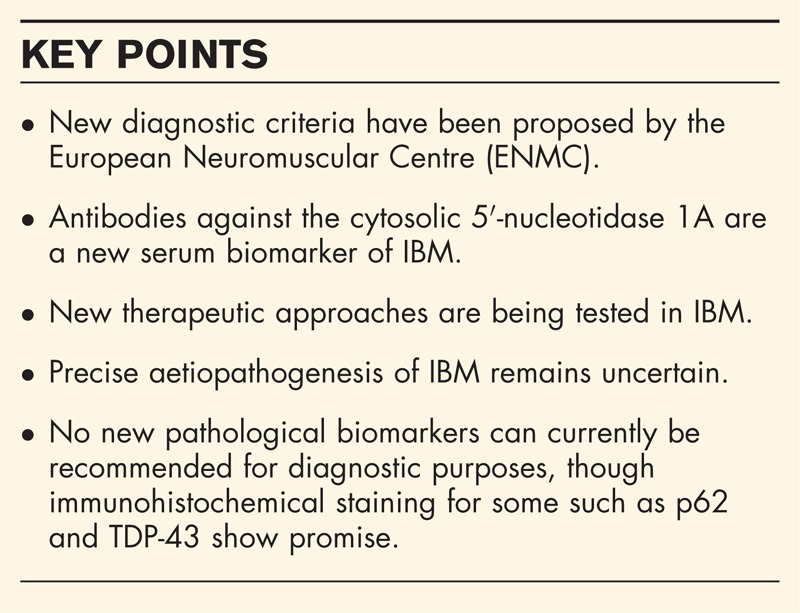
no caption available

Regarding prognostic factors, Benveniste *et al.*[3] found that male sex [hazard ratio 2.4, 95% confidence interval (95% CI) 1.5–3.9], older age (>60 years) (hazard ratio 2.0, 95% CI 1.3–3.1) and immunosuppressive treatment (hazard ratio 2.1, 95% CI 1.3–3.3) were predictive of progression of disease towards handicap for walking. However, once a walking aid was needed, progression towards the use of a wheelchair was not associated with these variables.

Cortese *et al.*[16^▪^] also found that older age (>55 years) at disease onset was predictive of a shorter time to requirement of a walking stick (hazard ratio 4.1, 95% CI 1.7–9.8), but not sex or treatment.

Prospective data in IBM are scarce and limited to small numbers of patients [15,16^▪^,^17^–^20^]. Mean decline in muscle strength by manual muscle testing was 3.5 ± 1.6% per year in the study by Cox *et al.*[17] and 5.2 ± 5.9% over 1 year in the study by Cortese *et al.*[16^▪^]. Quantitative muscle testing of quadriceps extensors and the IBM functional rating scale may be sensitive tools to monitor disease progression [15,16^▪^,^21^,^22^].

## HISTOPATHOLOGY

**FIGURE 1:**
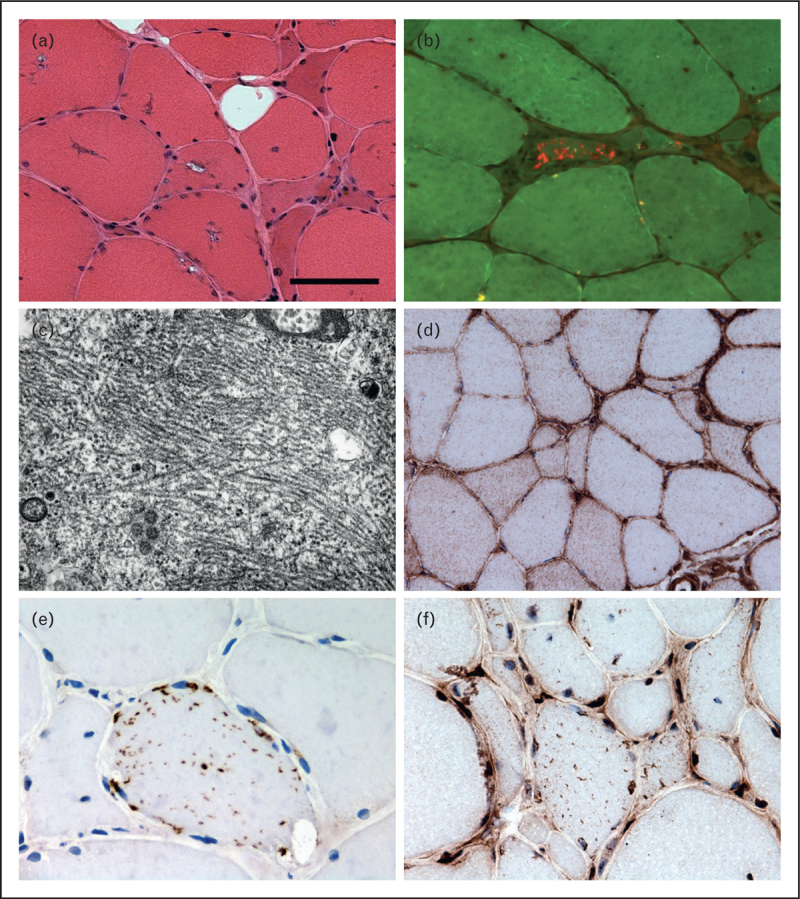
Pathological features observed in IBM. Muscle biopsy from a patient with IBM showing fibres containing rimmed vacuoles (a), amyloid in a tissue section stained using Congo red and visualised under fluorescent light (b) and tubulofilaments observed using electron microscopy (c). Immunohistochemically stained tissue sections reveal increased sarcolemmal and sarcoplasmic major histocompatibility complex class I (MHC Class I) expression (d) and fibres containing sarcoplasmic p62 immunoreactive aggregates (e) and TAR DNA-binding protein 43 (TDP-43) immunoreactive aggregates with loss of normal myonuclear TDP-43 staining (f). Scale bar in A represents 50 μm in (a), (b), (d), (f); 25 μm in (e); and 0.7 μm in (c).

Muscle biopsies from patients with IBM typically show several different pathological features, broadly described as inflammatory or degenerative. Haematoxylin and eosin (H&E) staining reveals fibre necrosis and regeneration, rounded atrophic fibres, split fibres and eosinophilic inclusions (Fig. 1). Evidence of neurogenic atrophy may sometimes be seen. Using more specialist techniques, a number of other pathological features have been described such as protein accumulations, increased major histocompatibility complex class I (MHC Class I) expression and mitochondrial changes [e.g. the presence of cytochrome c oxidase (COX) negative fibres]. Tubulofilaments visualized within fibres using electron microscopy were the first pathological abnormality associated with IBM [23]. Subsequently, rimmed vacuoles and amyloid were described [24,25]. Although all the diagnostic pathological features associated with IBM have all been documented in other myopathies, in combination they are still considered to be highly specific for IBM. However, clinical experience and studies [3,8] have shown that they lack sensitivity. Despite long-standing awareness of the presence of these pathological features in IBM, it remains unclear how they relate to disease pathogenesis.

More recently, using immunohistochemical techniques, many different proteins have been found accumulated in IBM, leading to its description as a ‘promiscuous proteinopathy’ [26]. The proteins described are associated with different cellular processes such as inflammation, autophagy and endoplasmic reticulum stress. Many proteins reported in IBM were originally described in neurodegeneration, leading some authors to identify similarities between the pathogenesis of IBM and neurodegenerative conditions such as Alzheimer's disease. However, the validity of some of these findings is uncertain and has been questioned by others [27]. Currently, none of the protein aggregates described in the literature can be clearly recommended for diagnostic use in IBM, though present evidence appears to favour p62 and TAR DNA-binding protein 43 (TDP-43) as potential biomarkers. The absence of partial invasion and COX-negative fibres appears to be good evidence against a diagnosis of IBM [28]. A comprehensive review of pathological biomarkers has been recently published [29].

## AETIOPATHOGENESIS

The aetiopathogenesis of IBM remains uncertain. The varied pathological findings observed have driven a number of theories, including viral infection, accumulation of toxic proteins, autoimmune attack, myonuclear degeneration, endoplasmic reticulum stress and impairment of autophagy and proteasomal proteolysis. In addition to inflammatory changes, much of the recent work has focused on myonuclear degeneration and autophagy.

Myonuclear abnormalities are not uncommon in IBM. The presence of nuclear and lysosomal proteins in rimmed vacuoles led to the hypothesis that they are derived from degenerating myonuclei [30–32]. Further evidence of myonuclear involvement in the pathogenesis of IBM is suggested by the loss of myonuclear TDP-43 [33,34] and the presence of myonuclear protein aggregates. Sarcoplasmic TDP-43 inclusions have been reported to be one of the most abundant protein aggregates in IBM, found in up to 23% of fibres, suggesting that TDP-43 redistribution may play a significant role in the pathogenesis [34]. There is some evidence that TDP-43 loss from the myonuclei leads to abnormalities in the morphology of nuclei and apoptosis [35]. However, sarcoplasmic TDP-43 aggregates are not specific to IBM [33,36,37] and other publications have not reported such abundant changes [38].

Autophagy is responsible for the degradation of long-lived cytosolic proteins and organelles. Initially, it was thought to be an indiscriminate process; however, there is increasing evidence of its selectivity [39]. Impairment of autophagy leads to the accumulation of p62 [40,41]. p62 and a number of other autophagy-associated proteins, including LC3 and neighbour of BRCA 1 gene 1 (NBR1), have been found to be increased in IBM [38,42,43]. Whether this reflects impairment of autophagic degradation or increased autophagic turnover is unknown. In addition to degrading and recycling cellular organelles such as mitochondria, autophagy may also affect MHC Class I turnover. Therefore, abnormalities in this pathway may explain several of the varied pathological features observed in IBM.

## DIAGNOSTIC CRITERIA

The first diagnostic criteria for IBM were proposed by Calabrese *et al.*[44]. These required the presence of microtubular filaments in inclusions and rimmed vacuoles for a diagnosis of definite IBM and probable IBM respectively, reflecting the belief that these pathological findings were sensitive and specific for IBM. Further criteria were proposed by Lotz *et al.*[5]. They found that rimmed vacuoles, atrophic fibres, endomysial autoaggressive inflammatory exudate and tubulofilaments were essential pathological features for a diagnosis of IBM. However, these criteria were based solely on the analysis of patients with rimmed vacuoles on muscle biopsy.

The seminal Griggs criteria were published in 1995 (Table 1) [4]. These are similar to the two previous criteria and therefore pathologically focused. Using the Griggs criteria, a diagnosis of definite IBM could be made on the basis of the pathological findings alone. In the absence of tubulofilaments and amyloid, a diagnosis of possible IBM could be made but required additional clinical and laboratory criteria to be satisfied. Amendments to the criteria, including the assessment of mitochondrial changes and MHC Class I upregulation, have been suggested but not widely adopted [45]. The first ENMC diagnostic criteria were published in 1997 [7]. A significant change was the ability to make the diagnosis in the absence of rimmed vacuoles and tubulofilaments (Table 2) [46].

It is now recognized that, although the pathological findings are highly specific when present in combination, they lack sensitivity. However, the combination of selective weakness of finger flexion and knee extension is believed to be typical of IBM and not present in other myopathies. To address this, more recent criteria [10,11], including the 2011 ENMC diagnostic criteria (Table 3) [9,47], include a category of clinically defined IBM.

## AUTOANTIBODIES TO CYTOSOLIC 5′-NUCLEOTIDASE 1A

In 2011, Salajegheh *et al.*[12] reported an autoantibody against a 43-kDa muscle antigen highly specific for IBM. This autoantibody was recently identified by the same group as targeting the cN1A [13^▪▪^]. The authors have also shown that in IBM muscle sections stained with a commercial anti-cN1A antibody, immunoreactivity was predominantly located to perinuclear regions and rimmed vacuoles [13^▪▪^].

Simultaneously, an independent European group reported an autoantibody against a 44-kDa muscle antigen. This antibody was named anti-Mup44 and its target was identified as also being the cN1A [14^▪▪^]. The antibodies identified by these two groups are therefore targeting the same antigen and the very small difference in molecular weights (43 versus 44 kDa) is probably related to methodological variation between laboratories [13^▪▪^,^14^^▪▪^].

The diagnostic performance of anti-cN1A reactivity was very good. Results were consistent across the two studies [13^▪▪^,^14^^▪▪^], with sensitivities of 60–70% and specificities of 83–92% for low antibody titres, and sensitivities of 33–34% and specificities of 96–98% for high antibody titres (Table 4). This new antibody has therefore significant potential utility in clinical practice and re-launches the debate about the role of autoimmunity in IBM pathogenesis.

## IMAGING

MRI is becoming increasingly important in myositis and neuromuscular diseases in general. Its role in the diagnosis and management of inherited muscle diseases and inflammatory myopathies, including IBM, has recently been comprehensively reviewed [48,49].

Qualitative conventional MRI techniques have mainly been used to define disease-specific patterns of muscle involvement. MRI can also be useful to monitor disease progression and response to treatment, or to direct the muscle biopsy, especially in inflammatory muscle diseases.

**FIGURE 2:**
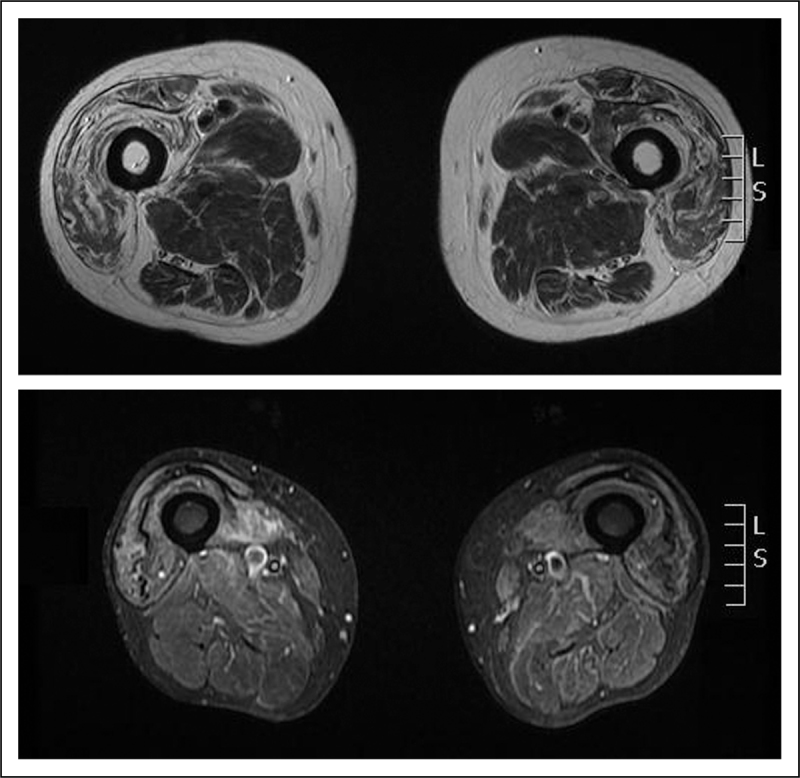
Transverse T1-weighted (upper slice) and STIR image (bottom slice) of the thighs of patients with inclusion body myositis. Upper row: note the fatty infiltration (areas of increased signal) predominantly of the anterior muscles of the thigh. Bottom row: note the areas of high signal in the right thigh (also in the anterior muscles), indicating muscle oedema (inflammation).

T1-weighted sequences are usually used to detect chronic muscle disease (fatty infiltration). The short tau inversion recovery (STIR) sequence is usually used to detect acute pathology (inflammation) (Fig. 2). Consistently with previous observations, in a cohort of 32 IBM patients, Cox *et al.*[50] recently reported that muscle inflammation was less common than fatty infiltration in IBM and that the number of muscles infiltrated with fat correlated with weakness and disability. Fatty infiltration was more frequently observed in the deep finger flexors, anterior muscles of the tights (often with relative sparing of the *rectus femoris*) and all the muscles of the lower leg, particularly the medial part of the gastrocnemius. There was no disease control group in this study, which limits its interpretation. Patchy areas of muscle inflammation or proximal involvement can be suggestive of both polymyositis and dermatomyositis, while myofascial oedema or a reticular subcutaneous pattern is more typical of dermatomyositis [51].

Quantitative MRI techniques such as the three-point Dixon fat-water quantification, T1-relaxometry, T2-relaxometry and magnetization transfer imaging are currently being evaluated in IBM and other muscle diseases and may prove to be reliable and sensitive outcome measures for clinical trials and observational studies [48,49].

Increased Pittsburgh Compound B [a PET biomarker that detects amyloid β) uptake levels in the gastrocnemius muscle have recently been described in seven IBM patients (compared with six non-IBM patients) [52]. Larger studies with this PET biomarker are needed to confirm these results and to clarify the potential utility of Pittsburgh Compound B in clinical practice and in the research setting.

## TREATMENT

Evidence-based treatment recommendations cannot be made in IBM and the limited studies [3,53^▪^] so far have shown that the disease is resistant to immunosuppressive drugs. A recent retrospective study [54] in 16 IBM patients suggested short-term benefit of intravenous immunoglobulin (IVIg) treatment on muscle strength and dysphagia. However, this benefit was only temporary and limited to a small proportion of patients. The role of IVIg in the treatment of IBM is yet to be clarified in an adequately powered randomized controlled trial (RCT).

The effects of exercise in patients with inflammatory myopathies, including IBM, have been recently reviewed [55^▪^]. There are promising data from open-label studies, but larger RCTs are needed to evaluate the possible effect of exercise in IBM [55^▪^]. A randomized cross-over trial (*n* = 30) aimed at investigating the effect of aerobic training in IBM is currently recruiting patients [56].

Other potential new therapeutic agents are being investigated in early phase studies. Modulating the cytoprotective heat shock response (HSR) represents a therapeutic strategy through which the detrimental aspects of both inflammation and degeneration could be dampened. A placebo-controlled trial with arimoclomol (16 active drug, eight placebo), an orally administered pharmacological agent that can upregulate the HSR by amplifying heat shock protein expression, was recently reported in abstract format. Arimoclomol was well tolerated and demonstrated a preliminary signal for potential therapeutic benefit in patients with IBM, supporting further research of arimoclomol in this disease [57].

Antagonists of myostatin could potentially be used as therapeutic agents in IBM. Myostatin is a protein that negatively regulates skeletal muscle growth, and myostatin antagonists have shown promise for increasing muscle mass and strength in animal studies. The myostatin pathway is currently being investigated in two studies. A placebo-controlled trial (11 active, three placebo) with BYM338, an intravenously administered mAb that binds competitively to activin receptor type IIB with greater affinity than myostatin, was recently completed and results are awaited [58]. Intramuscular follistatin gene transfer (follistatin is a naturally occurring antagonist of myostatin) is also being tested in an open-trial enrolling nine IBM patients [59].

Etanercept, a tumour necrosis factor (TNF) antagonist administered subcutaneously, is currently being tested in a placebo-controlled study [60] with 30 patients. Results from one open-label study [61] evaluating 20 patients treated with lithium are also expected; animal studies have shown that lithium can modulate tau phosphorylation via suppression of glycogen synthase kinase-3β.

## CONCLUSION

IBM is still an enigmatic and often misdiagnosed disease. The pathogenesis of the disease is not fully understood and pharmacological treatments have failed to show efficacy. However, recent advances and the increasing efforts of the scientific community to disentangle the disease mechanisms allow us to be optimistic about the future. New diagnostic criteria have been proposed by the ENMC, reflecting the knowledge that typical pathological findings may be absent in patients with clinically typical IBM. The new anti-cN1A antibody represents an important advance that may help early diagnosis in clinical practice. The role of MRI in IBM is expanding, not only as a diagnostic tool but also as a potential outcome measure in clinical trials. New therapeutic avenues are being explored and some of these may progress into efficacy trials. Future research should focus on increasing understanding of the pathophysiological mechanisms of the disease and on the identification of reliable and sensitive outcome measures for clinical trials. International collaboration will be particularly important to translate research advances into tangible patient benefits and improved patient outcomes.

## Acknowledgements

M.G.H. is supported by an MRC Centre grant 2013–2018 (MR/K000608/01). S.B. is supported by the Myositis Support Group. Research undertaken by the authors is supported by the UCLH NIHR Biomedical Research Centre. We would like to thank Dr Janice Holton and Dr Matt Parton for critically reviewing the manuscript and Ms Kerrie Venner (Technical Manager, Electron Microscopy Unit, UCL Institute of Neurology) for her technical assistance.

### Conflicts of interest

The authors have no competing interests.

## REFERENCES AND RECOMMENDED READING

Papers of particular interest, published within the annual period of review, have been highlighted as:

▪ of special interest
▪▪ of outstanding interest

## Figures and Tables

**Table 1:** 1995 Griggs diagnostic criteria

Criteria type	Features
Clinical features	Duration of illness >6 months
	Age of onset >30 years old
	Muscle weakness affecting proximal and distal muscles of arms and legs and patient must exhibit at least one of the following features:
	Finger flexion weakness
	Wrist flexion weakness > wrist extension weakness
	Quadriceps muscle weakness (≤grade 4 MRC)
Laboratory features	Serum creatine kinase <12 times normal
	Muscle biopsy
	Inflammatory myopathy characterixed by mononuclear cell invasion of nonnecrotic muscle fibres
	Vacuolated muscle fibres
	Either
	Intracellular amyloid
	15–18 nm tubulofilaments
	Electromyography must be consistent with features of an inflammatory myopathy
Definite IBM	Patients must exhibit all muscle biopsy features, including invasion of nonnecrotic fibres by mononuclear cells, vacuolated muscle fibres and intracellular (within muscle fibres) amyloid deposits or 15–18 nm tubulofilaments.
Possible IBM^a^	If the muscle biopsy shows only inflammation (invasion of nonnecrotic muscle fibres by mononuclear cells) without other pathological features of IBM, then a diagnosis of possible IBM can be given if the patient exhibits the characteristic clinical (1–3) and laboratory (4,6) features.

^a^In the text of the original article by Griggs *et al.* [4], possible IBM can be diagnosed if the muscle biopsy fails to show intracellular amyloid deposits and 15–18 nm tubulofilaments; therefore, an inflammatory infiltrate characterized by mononuclear cell invasion of nonnecrotic fibres and vacuolated muscle fibres are necessary features.

**Table 2:** 2007 European Neuromuscular Centre diagnostic criteria^a^

Criteria type	Features
Clinical	Presence of muscle weakness
	Weakness of forearm muscles, particularly finger flexors, or wrist flexors more than wrist extensors
	Slowly progressive course
	Sporadic disease
Histopathology	Mononuclear inflammatory infiltrates with invasion of nonnecrotic muscle fibres
	Rimmed vacuoles
	Ultrastructure: tubulofilaments of 16–21 nm
Definite IBM	1,2,3,4,5,6 or 1,3,4,5,6,7
Probable IBM	1,2,3,4,5 or 1,3,4,5,6

^a^Adapted from [46].

**Table 3:** 2011 European Neuromuscular Centre diagnostic criteria [9,47]

Clinical features	Classification	Pathological features
Duration of weakness >12 months	Clinicopathologically defined IBM	All of the following:
Creatine kinase ≤15× ULN		Endomysial inflammatory infiltrate
Age at onset >45 years		Rimmed vacuoles
Finger flexion weakness > shoulder abduction weakness		Protein accumulation^a^ or 15–18 nm filaments
AND/OR		
Knee extension weakness ≥ hip flexor weakness		
Duration of weakness >12 months	Clinically defined IBM	One or more, but not all, of:
Creatine kinase ≤15× ULN		Endomysial inflammatory infiltrate
Age at onset >45 years		Upregulation of MHC Class I
Finger flexion weakness > shoulder abduction weakness		Rimmed vacuoles
AND		Protein accumulation^a^ or 15–18 nm filaments
Knee extension weakness ≥ hip flexor weakness		
Duration of weakness >12 months	Probable IBM	One or more, but not all, of:
Creatine kinase ≤15 ULN		Endomysial inflammatory infiltrate
Age at onset >45 years		Upregulation of MHC Class I
Finger flexion weakness > shoulder abduction weakness		Rimmed vacuoles
OR		Protein accumulation^a^ or 15–18 nm filaments
Knee extension weakness ≥ hip flexor weakness		

Demonstration of amyloid or other protein accumulation by established methods (e.g. for amyloid Congo red, crystal violet, thioflavin T/S, for other proteins p62, SMI-31, TDP-43). MHC Class I, major histocompatibility complex class I; ULN, Upper limit of normal.

**Table 4:** Sensitivity and specificity (high and low antibody titres) of anti-cN1A antibodies for the diagnosis of IBM in the group of patients with neuromuscular diseases and in the subgroup of patients with inflammatory myopathies^a^^,^^b^

Study		High titre^c^		Low titre^c^	
	N-total (% IBM)	Sensitivity	Specificity	Sensitivity	Specificity
Patients with neuromuscular diseases (i.e. excluding healthy controls)					
Larman *et al.* [13^▪▪^]	165 (28%)	34%	98%	70%	92%
Pluk *et al.* [14^▪▪^]	234 (40%)	33%	96%	60%	89%
Subgroup of patients with inflammatory myopathies (IBM, PM, DM and IMNM)					
Larman *et al.* [13^▪▪^]	123 (38%)	34%	97%	70%	89%
Pluk *et al.* [14^▪▪^]	140 (67%)	33%	96%	60%	83%

cN1A, cytosolic 5́-nucleotidase 1A; DM, dermatomyositis; IBM, inclusion body myositis; IMNM, immune-mediated necrotizing myopathy; N-total (% IBM), total number of patients (percentage of IBM patients); PM, polymyositis.

^a^Study population in Larman *et al.* [13^▪▪^]: 47 patients with IBM, 26 with PM, 36 with DM, 14 with IMNM, 13 with myasthenia gravis, 4 with myotonic dystrophy, 4 with limb-girdle muscular dystrophy, 1 with myofibrillar myopathy, 1 with distal myopathy with rimmed vacuoles, 19 with other muscular diseases and 35 healthy controls.

^b^Study population in Pluk *et al.* [14^▪▪^]: 94 patients with IBM, 24 with DM, 22 with PM, 94 with other neuromuscular disorders and 32 healthy controls.

^c^High and low anticN1A titres (reactivities) were defined as >10 intensity units (IU) (scaled threshold based on the dot blot densitometry mean as well as 3 standard deviations for the 35 tested healthy individuals) and >2.5 IU, respectively, in the study by Larman *et al.* [13^▪▪^], and as >5 and >1% precipitation of the input cN1A protein, respectively, in the study by Pluk *et al.* [14^▪▪^].
